# Gender differences in aortic blood pressure, arterial stiffness, and cerebral blood flow in healthy adults: A STROBE-compliant cross-sectional observational study

**DOI:** 10.1097/MD.0000000000042717

**Published:** 2025-06-06

**Authors:** Salahaden R. Sultan

**Affiliations:** a Department of Radiologic Sciences, Faculty of Applied Medical Sciences, King Abdulaziz University, Jeddah, Saudi Arabia.

**Keywords:** aortic blood pressure, arterial stiffness, cardiovascular system, cerebral blood flow, gender differences

## Abstract

Understanding gender-specific differences due to anatomical and physiological variations in cardiovascular and cerebrovascular physiology is essential. Thus, this study explores variations in aortic and blood pressure, arterial stiffness, and cerebral blood flow between healthy men and women. The study involved 36 healthy participants (23 males and 13 females). Aortic blood pressure (aBP) and arterial stiffness were assessed using pulse wave analysis and pulse wave velocity, respectively, via Vicorder® software. Cerebral blood flow in the middle cerebral artery, internal carotid artery (ICA), and common carotid artery (CCA) was evaluated using high-resolution ultrasound, with automated edge-detection software used for ICA and CCA diameter measurements. Males showed significantly higher aBP and arterial stiffness compared to females (systolic aBP mean difference [MD] + 15.74 mm Hg, *P* < .001; diastolic aBP MD + 6.57 mm Hg, *P* = .01; arterial stiffness MD + 1.01 m/s, *P* < .001). In contrast, CCA peak systolic velocity (CCA-PSV) was significantly higher (MD + 0.20 m/s, *P* = .001) in males compared to females, with no significant differences were observed in middle cerebral artery-PSV (*P* = .10) nor ICA-PSV (*P* = .99). Males also had a larger CCA diameter (MD + 0.45 mm, *P* = .01) and ICA diameter (MD + 0.41 mm, *P* = .05). The findings of this study showed significant gender differences in aBP, arterial stiffness, and characteristics of the extracranial arteries. The observed of higher central BP and arterial stiffness, along with elevated CCA peak systolic velocity and larger CCA and ICA diameters combination in healthy males, suggests a compensatory mechanism that may help preserve cerebral perfusion in the presence of increased blood pressure and arterial stiffness. These highlight the importance of considering gender-specific cardiovascular and cerebrovascular responses in clinical assessments and interventions.

## 1. Introduction

Aortic blood pressure (aBP) and arterial stiffness are critical biomarkers of cardiovascular health, reflecting the functional integrity of the vascular system.^[1]^ The aorta sustain an optimal balance between pressure and elasticity to facilitate efficient hemodynamic function.^[2]^ Elevated aBP is indicative of increased hemodynamic load on the cardiovascular system, a condition often precipitating atherosclerosis, wherein lipid-rich plaques accumulate within the arterial walls, leading to luminal narrowing and a heightened risk of adverse cardiovascular events.^[3]^ Arterial stiffness, while frequently concomitant with aging and atherosclerosis, can also manifest independently due to alterations in the structural composition of the arterial wall, particularly in the content and configuration of collagen and elastin fibers.^[2]^ The loss of vascular elasticity exacerbates cardiovascular strain, thereby imposing significant risks on vital organs such as the brain and kidneys.^[4]^

Cerebral blood flow (CBF) is indispensable for maintaining neuronal viability and cognitive function, as it delivers essential oxygen and nutrients to the brain.^[5]^ Given the brain’s high metabolic demands, it is particularly vulnerable to disruptions in its blood supply, which can precipitate cognitive deficits and irreversible neuronal damage.^[6]^ Systemic blood pressure is a pivotal determinant of CBF, which is tightly regulated by cerebral autoregulation, a homeostatic mechanism that stabilizes cerebral perfusion despite fluctuations in systemic blood pressure.^[5]^ However, increased arterial stiffness can undermine this regulatory mechanism, leading to augmented transmission of pulsatile pressure into the cerebral circulation, thereby potentially damaging the microvasculature of the brain.^[7]^ This complex interplay between arterial stiffness and CBF underscores the intricate relationship between vascular and neurological health. Elevated arterial stiffness increases systemic blood pressure and compromises the brain’s autoregulatory capacity, thus elevating the risk of cerebral hypoperfusion and consequent cognitive decline.^[8,9]^ Furthermore, the progression of arterial stiffening is associated with an increased propensity for cerebrovascular events, such as stroke, due to its role in promoting.^[10,11]^

Gender-specific differences in cardiovascular and cerebrovascular function are factors influencing disease prevalence, progression, and outcomes. These differences are thought to arise from a complex interplay of anatomical, hormonal, and genetic factors that collectively influence hemodynamic responses and vascular health.^[12,13]^ Given this, we aimed to explore variations in aBP, arterial stiffness, and CBF between healthy men and women.

## 2. Methods

### 2.1. Study design and assessment

This pilot study was conducted in accordance with the Declaration of Helsinki and the International Conference on Harmonization of Good Clinical Practice guidelines, and obtain ethical approval from the Ethics Research Committee at the University of Nottingham (Reference No: E1411201, 34-1805). Healthy participants were recruited for the study, and written informed consent was obtained from all participants prior to enrollment. They were instructed to refrain from consuming vitamin supplements for 72 hours before the study visit and to avoid exercise, medications, caffeine, alcohol, and smoking for 24 hours prior. Measurements were performed in a room maintained at 22 to 24°C, following a minimum of 10 minutes of rest in the supine position after an overnight fast.^[14,15]^

### 2.2. Measurements of aBP, pulse wave velocity (PWV), and CBF

aBP was assessed through pulse wave analysis and arterial stiffness ws assessd through pulse wave velocity (PWV) between the carotid and femoral anatomical sites using Vicorder® software.^[16,17]^ Brachial blood pressure (bBP) was measured using sphygmomanometer. CBF was assessed in middle cerebral artery (MCA), common carotid artery (CCA) and internal carotid artery (ICA). For the MCA, low frequency transcranial Doppler probe (2 MHz) of Sonora transcranial Doppler system was used to detect changes in peak systolic velocity (PSV) through transtemporal acoustic window.^[18]^ CCA-PSV and ICA-PSV were measured using a high-frequency linear probe (L15-4 MHz) of a high-resolution ultrasound system (Terason 3200T). The carotid arteries diameter was determined using automated edge-detection software (Cardiovascular Suite Quipu).^[19,20]^ All measurements were performed by a single experienced operator to ensure consistency.

### 2.3. Statistical analysis

An independent samples *t* test was employed to assess differences between male and female participants. A level of ≤ 0.05 was considered statistically significance. The analysis was conducted using IBM SPSS Statistics version 21 (Armonk, NY: IBM Corp).

## 3. Results

A total of 36 individuals, including 26 males and 13 females were recruited for this study. The average age of participants was 26.67 ± 5.30 years. The overall mean body mass index was 24.19 ± 3.04. Systolic bBP averaged 124.56 ± 7.81 mm Hg, while diastolic bBP was 72.13 ± 4.35 mm Hg. Systolic aBP averaged 116.45 ± 11.23 mm Hg, while diastolic aBP was 71.13 ± 7.07 mm Hg. The mean cfPWV was 6.40 ± 0.83 m/s. The MCA-PSV was 0.78 ± 0.21 m/s, and the ICA-PSV was 0.81 ± 0.18 m/s. The ICA diameter measured 4.86 ± 0.60 mm, and the CCA-PSV was 0.90 ± 0.22 m/s. The CCA diameter was 6.95 ± 0.59 mm. Participant characteristics are summarized in Table 1.

**Table 1:** Participant characteristics.

Characteristics	Descriptive statistics (mean ± SD)		
	All (n = 36)	Male (n = 26)	Female (n = 13)
Age (years)	26.67 ± 5.30	26.83 ± 4.55	26.38 ± 6.62
BMI	24.19 ± 3.04	25.12 ± 1.91	22.55 ± 3.95
bBP systolic (mm Hg)	124.56 ± 7.81	128.74 ± 5.48	17.15 ± 5.47
bBP diastolic (mm Hg)	72.13 ± 4.35	73.78 ± 3.65	69.23 ± 4.06
aBP systolic (mm Hg)	116.45 ± 11.23	122.13 ± 8.50	106.38 ± 8.01
aBP diastolic (mm Hg)	71.13 ± 7.07	73.50 ± 5.90	66.92 ± 7.20
cfPWV (m/s)	6.40 ± 0.83	6.77 ± 0.77	5.75 ± 0.45
MCA-PSV (m/s)	0.78 ± 0.21	0.74 ± 0.19	0.86 ± 0.22
ICA-PSV (m/s)	0.81 ± 0.18	0.81 ± 0.19	0.80 ± 0.15
ICA-D (mm)	4.86 ± 0.60	5.00 ± 0.58	4.59 ± 0.57
CCA-PSV (m/s)	0.90 ± 0.22	0.97 ± 0.23	0.77 ± 0.21
CCA-D (mm)	6.95 ± 0.59	7.12 ± 0.63	6.66 ± 0.40

aBP = blood pressure, bBP = brachial blood pressure, BMI = body mass index, CCA = common carotid artery, cfPWV = carotid-femoral pulse wave velocity, D = diameter, ICA = internal carotid artery, MCA = middle cerebral artery, mmHg, PSV = peak systolic velocity, SD = standard deviation.

### 3.1. Aortic and bBP and arterial stiffness

**Figure 1.**
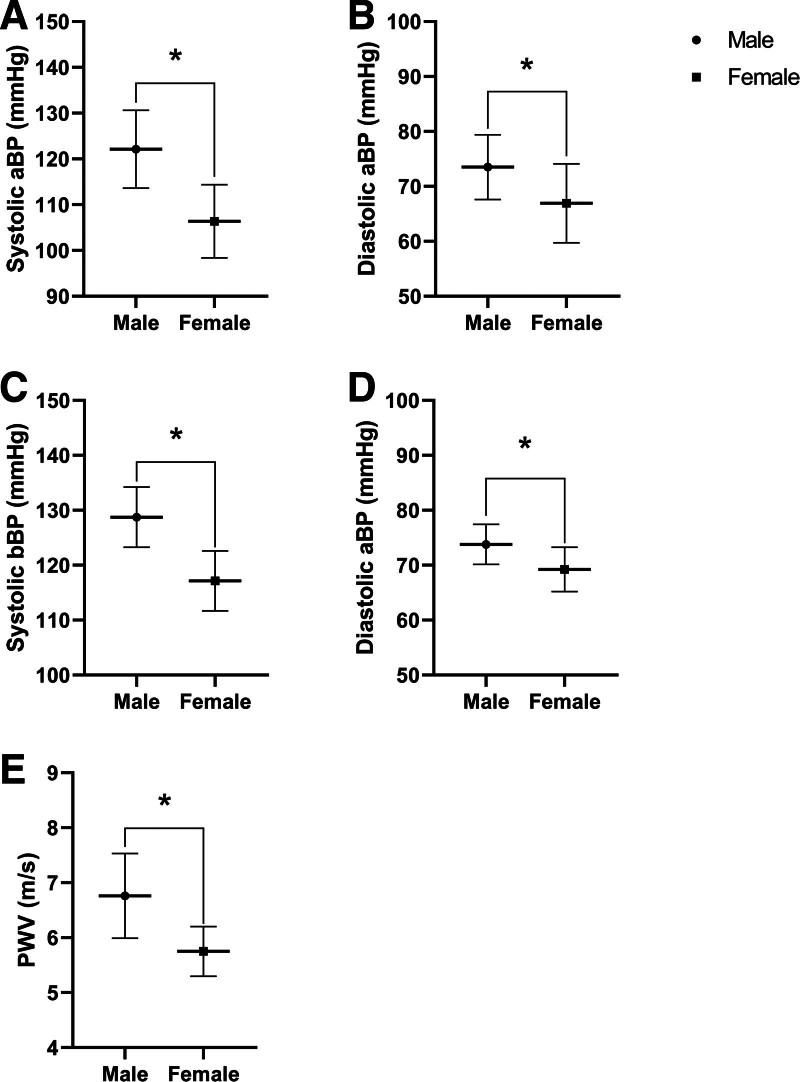
Differences in aortic blood pressure (aBP), brachial blood pressure (bBP), and arterial stiffness between males and females. Systolic aBP (A), diastolic aBP (B), systolic bBP (C), diastolic bBP (D), arterial stiffness through pulse wave velocity (PWV, E). *P ≤ .05 using independent *t* test.

Males had significantly higher aBP and bBP compared to females. For aBP, the mean systolic aBP difference was + 15.74 mm Hg (95% confident interval (CI) 9.90–21.59, *P* < .001, Fig. 1A) and the mean diastolic aBP difference of + 6.57 mm Hg (95% CI 1.69–11.45, *P* = .01, Fig. 1B). For bBP, the mean systolic bBP difference was + 11.58 mm Hg (95% CI 7.71–15.45, *P* < .001, Fig. 1C) and a mean diastolic aBP difference of + 4.55 mm Hg (95% CI 1.86–7.23, *P* = .02, Fig. 1D). In addition, arterial stiffness was greater in males, with a mean difference of + 1.01 m/s (95% CI 0.59–1.42, *P* < .001, Fig. 1E).

### 3.2. CBF

**Figure 2.**
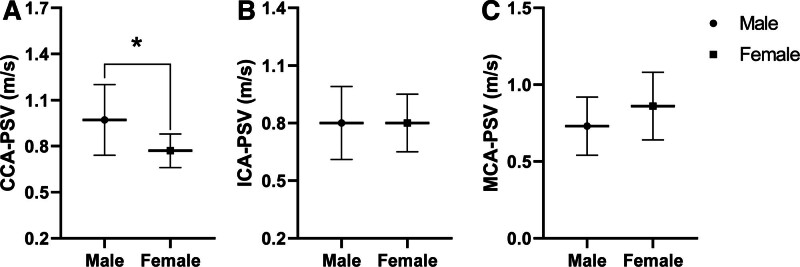
Differences in cerebral blood flow velocities between males and females. Common carotid artery peak systolic velocity (CCA-PSV, A), internal carotid artery peak systolic velocity (ICA-PSV, B), and middle cerebral artery peak systolic velocity (MCA-PSV, C) (mean ± SD). *P ≤ .05 using independent *t* test.

**Figure 3.**
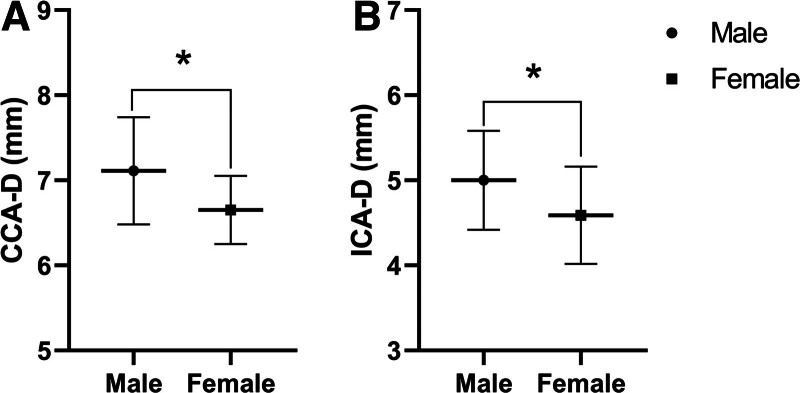
Differences in common carotid artery diameter (CCA-D, A) and internal carotid artery diameter (ICA-D, B) between males and females. *P ≤ .05 using independent *t* test.

CCA-PSV was significantly higher in males than females, with a mean difference of + 0.20 m/s (95% CI, 0.08–0.32, *P* = .001, Fig. 2A). No significant differences were observed in ICA-PSV (mean difference 0.0006, 95% CI, −0.12 to 0.12, *P* = .99, Fig. 2B) nor MCA-PSV (mean difference 0.12, 95% CI, −0.28 to 0.03, *P* = .11, Fig. 2C) between males and females. Males had a larger CCA diameter, with a mean difference of + 0.45 mm (95% CI, 0.10–0.81, *P* = .01, Fig. 3A), and ICA diameter, with a mean difference of + 0.41 mm, (95% CI, 0.0002–0.82, *P* = .05, Fig. 3B).

## 4. Discussion

This study provides a comprehensive analysis of gender differences in aBP, arterial stiffness, and CBF among healthy adults, showing a significant gender differences in in aBP, arterial stiffness, and the diameter and blood flow in extracranial arteries. These findings highlight the critical importance of recognizing these differences, given their profound implications for the diagnosis, prevention, and management of cardiovascular and cerebrovascular diseases.

Our findings demonstrate that males exhibit significantly higher arterial stiffness and blood pressure compared to females, consistent with the broader literature, which consistently shows that males tend to have elevated blood pressure levels.^[1]^ It has been reported that gender-based disparities in central and peripheral arterial pressure waveforms persist consistently across the lifespan, however, with advancing age, these disparities diminish, and the amplification observed is reduced to a minimal level.^[21]^ Several factors contribute to these observed differences, including variations in body composition, hormonal influences, and vascular function.^[1,12]^ For example, women typically have smaller heart sizes and stronger ventricular contractility than men,^[21]^ while female hormones exert protective effects on the cardiovascular system by enhancing endothelial function, promoting vasodilation, and reducing arterial stiffness.^[22]^ However, these hormones may also negatively impact vessel compliance.^[23]^ The implications of increased arterial stiffness in males are profound, including elevated blood pressure, which is correlated with increased blood pressure itself.^[11]^ Additionally, increased arterial stiffness imposes greater left ventricular afterload, requiring the heart to exert more effort to pump blood against the stiffer arteries, potentially leading to left ventricular hypertrophy and an increase in heart size.^[11]^ Previous studies also confirm that males generally exhibit greater arterial stiffness, likely due to differences in the structural components of the arterial wall, such as collagen and elastin, as well as the influence of sex hormones.^[24]^ For instance, hormones in females, particularly in premenopausal women, are believed to offer some protection against arterial stiffness, largely due to the beneficial effects of estrogen on vascular function.^[22,25]^ The increased aBP observed in males can be attributed to several physiological mechanisms. Males typically display heightened sympathetic nervous system activity, leading to increased heart rate and vascular resistance, which subsequently elevates blood pressure. Furthermore, they generally have a higher cardiac output due to larger heart size and greater muscle mass, along with higher peripheral vascular resistance, all contributing to elevated blood pressure levels.^[1]^

CBF is vital for maintaining brain function, as it delivers the oxygen and nutrients necessary for neuronal activity. Our study found that males exhibit higher CCA-PSV, and larger CCA and ICA diameter compared to females, suggesting that the extracranial arteries in males may compensate for the increased aBP and arterial stiffness observed in this group. This compensatory mechanism is likely crucial for preserving cerebral perfusion despite the systemic hemodynamic challenges posed by higher blood pressure and arterial stiffness. In the present study, we found no significant differences were observed between males and females in ICA-PSV or MCA-PSV. This finding suggests that cerebral autoregulation, the ability of cerebral blood vessels to maintain stable blood flow despite changes in systemic blood pressure, may be equally effective in both genders under healthy condition. However, gender differences in cerebral autoregulation have been reported in which females had higher autoregulatory indices and lower transfer function gains in the autoregulatory frequency band, indicating more effective maintenance of CBF in response to blood pressure fluctuations.^[26]^ The enhanced cerebral autoregulation observed in females could be due to several factors, including smaller vessel diameters, higher vascular reactivity, and the influence of sex hormones such as estrogen. Estrogen has been shown to improve vascular reactivity by enhancing the production of nitric oxide, a potent vasodilator that helps maintain CBF.^[27]^ These mechanisms may contribute to the better autoregulatory capacity in females, potentially protecting them from cerebrovascular events. However, it is important to note that the protective effects of estrogen diminish after menopause, leading to a decline in arterial compliance and an increased risk of cerebrovascular events in older women. This highlights the importance of monitoring and managing arterial stiffness and blood pressure in postmenopausal women to mitigate the risk of stroke and other cerebrovascular complications.^[28,29]^

A limitation of this study is the relatively small sample size, which was restricted to healthy young adults. As this was designed as a pilot study, the primary aim was to explore feasibility and provide preliminary data on gender-specific differences in cardiovascular and cerebrovascular parameters. Additionally, potential confounding factors, such as age, hormonal status, and lifestyle habits, socioeconomic background, were not assessed, which could influence the results. The observed gender imbalance (23 males vs 13 females) is also acknowledged as a limitation that may affect generalizability. Future research should involve larger, more diverse, and gender-balanced cohorts and account for these variables to provide a more comprehensive understanding of gender differences in cardiovascular and cerebrovascular parameters.

## 5. Conclusion

This pilot study highlights significant gender differences in cardiovascular and cerebrovascular physiology among healthy adults, revealing that males exhibit higher aBP, greater arterial stiffness, and larger common carotid artery and ICA diameters, along with higher common carotid artery PSV compared to females. Although preliminary due to the pilot nature and sample size, these findings provide valuable insight into gender-specific vascular physiology and may serve as a foundation for hypothesis-driven research. These findings suggest that males may rely on compensatory mechanisms to maintain cerebral perfusion despite increased blood pressure and arterial stiffness. The results emphasize the importance of considering gender-specific differences in clinical assessments and interventions for cardiovascular and cerebrovascular health, and highlight the need for future studies with larger and more representative samples to validate and expand upon these findings.

## Author contributions

**Conceptualization:** Salahaden R. Sultan.

**Data curation:** Salahaden R. Sultan.

**Formal analysis:** Salahaden R. Sultan.

**Methodology:** Salahaden R. Sultan.

**Writing – original draft:** Salahaden R. Sultan.

**Writing – review & editing:** Salahaden R. Sultan.
